# Concentration and pollution assessment of heavy metals within surface sediments of the Raohe Basin, China

**DOI:** 10.1038/s41598-019-49724-7

**Published:** 2019-09-11

**Authors:** Jin Wei, Maoqing Duan, Yiping Li, Amechi S. Nwankwegu, Yong Ji, Jie Zhang

**Affiliations:** 1 Key Laboratory of Integrated Regulation and Resource Development on Shallow Lakes, Ministry of Education, College of Environment, Hohai University, Nanjing, 210098 China; 2 The Department of Water Environment, China Institute of Water Resources and Hydropower Research, Beijing, 100038 China; 3 School of Hydraulic and Ecological Engineering, Nanchang Institute of Technology, Nanchang, 330099 China

**Keywords:** Environmental chemistry, Environmental impact

## Abstract

Surface sediment samples were collected from four areas (the Jingdezhen Industrialized Area (JDZ), Upstream (UP), the Dexing Mining Area (DX), and Downstream (DM)) to investigate the concentration and chemical composition of heavy metals. The sediments were analysed for Cu, Zn, Pb, Cd, Cr, As, and Ni using a sequential extraction scheme according to the improved BCR (European Community Bureau of Reference) method. The obtained results show that the maximum values of Cu (793.52 μg·g^−1^), Zn (72.09 μg·g^−1^), Pb (222.19 μg·g^−1^), and Cd (1.60 μg·g^−1^) were collected from the DX sampling area, while the JDZ area had the highest concentrations of Cr (97.09 μg·g^−1^), As (318.05 μg·g^−1^), and Ni (66.35 μg·g^−1^). The majority of metal values far exceeded their corresponding background values. The risk analysis of geo-accumulation index (*I*_*geo*_) indicated that the heavy metals Cu and As were the main pollution factors and each element of the pollution degree followed the order of: Cu > As > Pb > Cd > Cr > Zn. Metal partitioning characteristics were also considered and more than 80% of metals show potential bioavailability and toxic effects.

## Introduction

Heavy metals in soils are difficult to migrate due to their long residual time, strong concealment, toxicity, and other characteristics. Consequently, they may be absorbed by crops, enter the food chain, or migrate into water and atmosphere, thus threatening the health and reproduction of humans and animals^1,2^. Therefore, the treatment of heavy metal pollution in river sediments and soils has become a hot and challenging research topic.

Heavy metal toxicity is not only related to the total concentration of heavy metals, but also to the distribution of its speciation. Different forms exert different environmental effects, which directly affects the toxicity of heavy metals, their migration, and natural cycling^3^. With regard to the form of heavy metals, no uniform definition and classification have been reported. The following heavy metal speciation analysis in soil and sediments are available: Tessier *et al*.^4^ divided the heavy metal forms in sediments or soils into exchangeable fraction, carbonate fraction, Fe-Mn oxide fraction, organic fraction, and residual fraction. Gambrell^5^ suggested that there are seven types of shape states of heavy metals in soils and sediments. These are water soluble fraction, easily exchangeable fraction, inorganic compounds precipitate fraction, macromolecule humus fraction, hydroxide precipitation absorption or adsorption fraction, sulfide precipitation fraction, and residual fraction. Shuman^6^ divided the heavy metals into exchangeable fraction, water soluble fraction, carbonate fraction, loose binding organic fraction, manganese oxide fraction, tight binding organic fraction, amorphous iron oxide fraction, and silicate minerals fraction. For the integration of these various classifications and methods, European Community Bureau of Reference proposed the BCR method, divided the heavy metals into four types of genera, namely acid soluble (such as carbonate fraction), reducible fraction (such as Fe-Mn oxide fraction), oxidizable fraction (such as organic fraction), and residual fraction. This is called the BCR extraction method.

Poyang Lake is China’s largest freshwater lake and one of the most important wetlands in the world with a complex ecological diversity and biological resources. Raohe Basin is one of the five major watersheds in Poyang Lake and is affected by its upstream Dexing copper mining and other heavy industrial pollution. Consequently, the soil and water environment has been polluted at different levels. In particular, the heavy metals Cu, Zn, Pb, and Cd cause more prominent pollution problems for the watershed, which have impacted Raohe and the rural ecological environment of the Poyang Lake region^7^. Therefore, it is necessary to investigate the spatial distribution of heavy metals in sediments from Raohe and assess the risk caused by these heavy metals to protect the corresponding aquatic ecosystem.

## Materials and Methods

### Study area and sampling sites

**Figure 1:**
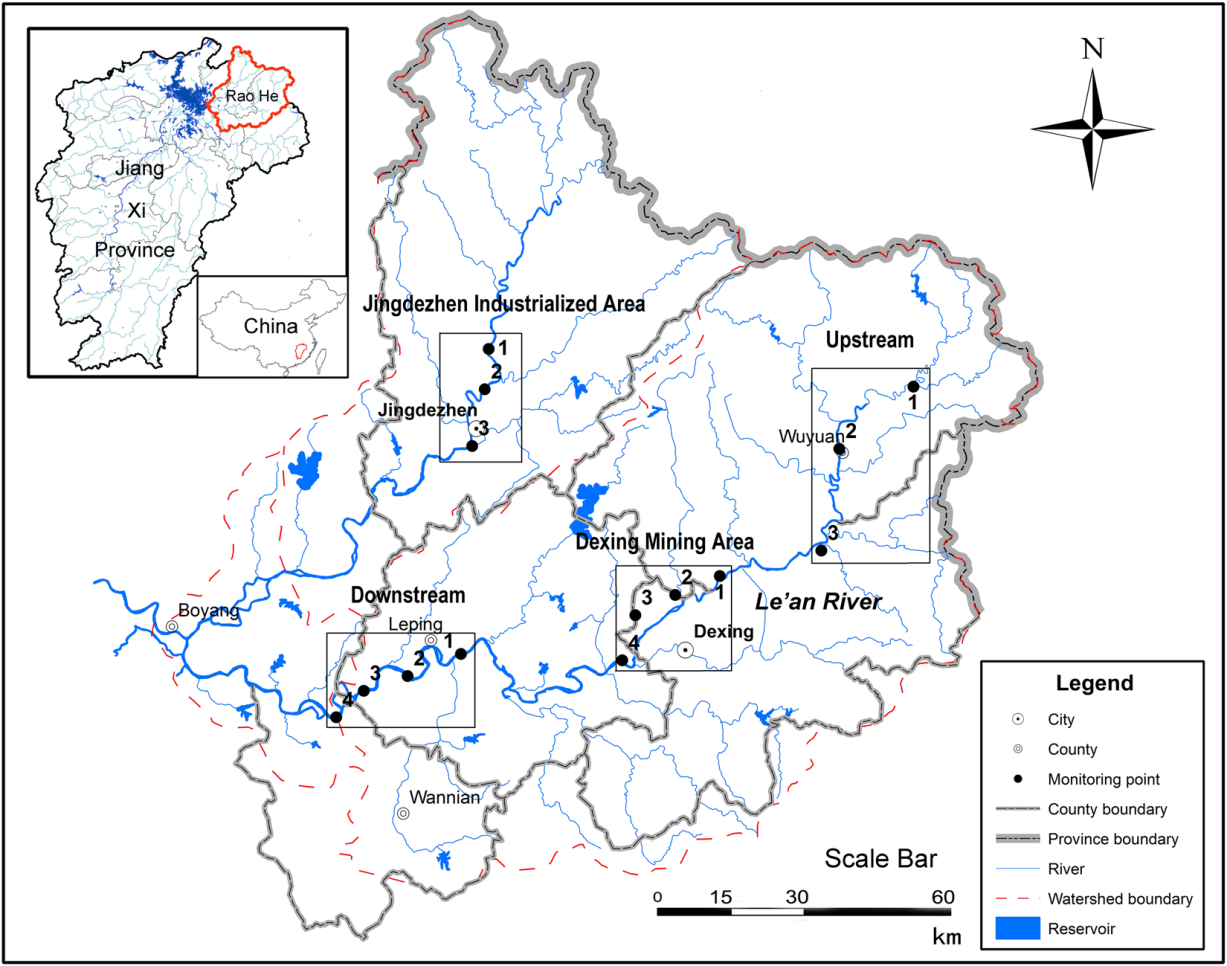
Research area and the geographical location of sampling sites.

The Raohe Basin has two main streams: Changjiang River and Le’an River. Changjiang River flows through the whole territory of Jingdezhen City from north to south. As a world-famous city of the ceramic industry, Jingdezhen City has many industrial parks, which affect the Changjiang River water environment. The other main stream, the Le’an River, runs by three large mines since the 1950s, including Asia’s largest copper mine (the Dexing Copper Mine). These mines account for more than 10% of the sewage flow of the river. The study was carried out along the Raohe Basin’s trunk streams. Based on the pollution and topographic characteristics, the Le’an River was divided into three partitions. A total of four typical areas were selected to indicate the whole region: the Jingdezhen Industrialized Area (JDZ), Upstream (UP), the Dexing Mining Area (DX), and Downstream (DM). The sampling points are presented in Fig. 1.

### Sampling and assay

The field sampling was conducted within the period from October to November, 2015 in these four areas. All samples were extracted from top horizon sediments (0–20 cm depth), and three or four samples were collected and pooled at each sampling point. Samples were air-dried, sifted, and then dried (105 °C) again prior to assays.

After freeze drying and homogenization, 0.5 g was weighed and placed in the digestion tank. HCl (3 ml), HNO_3_ (1 ml), and HClO_4_ (1 ml) were added and the digestion tank was then moved into the MLS-1200 MEG high-performance microwave digester. Digestion lasted for 30 mins at 150 °C. The sample solution was cooled and transferred to a 50 ml volumetric flask. HNO_3_ (1 ml) and distilled water were added for the analysis of total concentration of heavy metals. An aliquot of the sample was used for heavy metal partitioning analysis following the BCR sequential extraction method^8,9^. Briefly, 0.11 M HOAc (40 ml) was added to 1 g sediment sample for the acid soluble. Shaking was continued for 16 hours at 22 °C, causing the extract to separate via centrifugation (20 min) at 3000 × g. Yielding reducible fraction: The residue was treated with 0.5 M NH_2_OH·HCl (40 ml, PH 2) before shaking and separation. Hydrogen peroxide (H_2_O_2_) (10 ml, PH 2–3) was added to the above residue at room temperature, then heated to 85 ± 2 °C for 60 mins. The process was repeated before 1 M NH_4_OAc (50 ml, PH 2) was further added following shaking which lasted for 16 hours at 22 °C. The extract was separated via centrifugation and an oxidizable fraction was obtained. Distilled H_2_O (3 ml), 6 M HCl (7.5 ml), and 14 M HNO_3_ (2.5 ml) were later added to the above residue, left standing overnight at 20 °C, then boiled under reflux for 2 hours. This was finally cooled, filtered and a residual fraction obtained. The difference of total metal concentration with the above methods was <10%.

Prior to use, all the glass vessels were soaked with diluted HNO_3_ (15%), followed by repeated rinsing using de-ionized water^10^. Inductively coupled plasma-atomic emission spectrometry (ICP-AES) was equipped to measure the concentrations of heavy metals. For each group of analytical samples, two spiked blanks and two method blanks were simultaneously processed. The regression coefficient of calibration standards for different metals was higher than 0.999. The relative standard deviations (RSD) of these elements were calculated and presented in Table 1, which indicated a high accuracy of methods.

**Table 1:** Relative standard deviations (RSD) for different elements.

	Cu	Zn	Pb	Cd	Cr	As	Ni
RSD	4.9%	2.8%	3.3%	4.8%	4.3%	4.0%	2.1%

### Evaluation of the contamination degree

#### Geo-accumulation Index

The geo-accumulation index (*I*_*geo*_) is a common standard with which to evaluate the pollution of heavy metal in sediments^11^. *I*_*geo*_ is calculated by computing the base 2 logarithm of the measured total concentration of the metal over its background concentration using the following equation:

1 $${I}_{geo}={\log }_{2}({C}_{n}/1.5{B}_{n})$$

Where *C*_*n*_ represents the measured concentrations of heavy metals and *B*_*n*_ represents the geo-chemical background concentrations of the metals. Factor 1.5 was used as background value of lithological variability^12,13^.

#### Sediment quality values (SQVs)

Sediment quality values were designed to assist in interpreting the sediment quality and assessing the impact of sediment pollution on aquatic organisms^14^. The screening quick reference table (SQUIRT) are hereby introduced to evaluate whether the heavy metals measured in the Raohe Basin would breach these thresholds. The guideline values are subdivided into five increasing categories of remarkable effects, which have been derived from using different approaches. Sediment guidelines comprise threshold effect level (TEL), effect range low (ERL), probable effect level (PEL), effect range median (ERM), and apparent effect threshold (AET)^15,16^.

## Results and Discussion

### Heavy metal concentrations

Concentrations of heavy metals (Cu, Zn, Pb, Cd, Cr, As, and Ni) for JDZ, UP, DX, and DM are presented in Table 2. The range and averaged values of heavy metal concentrations (mg·kg^−1^) in surface sediments are: Cu, 15.58–793.52 (197.21); Zn, 11.21–72.09 (32.31); Pb, 14.28–222.19 (39.63); Cd, 0.00–1.60 (0.51); Cr, 13.08–97.09 (35.26); As, 12.87–318.05 (78.52); Ni, 18.38–66.35 (31.03). These data obtained from different areas of the Raohe Basin show considerable spatial variability. The highest concentrations of heavy metals were generally made out in mud flats where surface sediments were located close to heavily polluted industrial areas and mining industry areas in JDZ and DX. This spatial variability is caused by different proximities to contaminant sources^17^, and the considerable spatial variability indicates the strong impact of human activities on the presence of heavy metal, namely, mining activities, the petroleum industry, and internal sewage runoff. It is generally believed that aquatic ecosystem could be polluted with heavy metals enriched in various ways, in which human interference is the main reason that increases the content of trace metals dumped into water^18^.

**Table 2:** Concentration of metals observed in sediments collected from JDZ, UP, DX, and DM.

	Cu	Zn	Pb	Cd	Cr	As	Ni
JDZ (μg·g^−1^)	101.26 ± 7.66	34.12 ± 1.49	23.76 ± 1.04	1.05 ± 0.11	97.09 ± 2.39	95.13 ± 3.33	66.35 ± 1.44
	59.81 ± 3.42	33.44 ± 0.91	27.60 ± 1.40	0.23 ± 0.03	33.70 ± 1.00	120.48 ± 5.79	55.55 ± 0.56
	15.58 ± 1.21	52.92 ± 1.33	16.13 ± 0.55	0.00	24.10 ± 0.87	318.05 ± 4.99	45.60 ± 0.66
UP (μg·g^−1^)	36.52 ± 4.11	21.17 ± 1.00	23.01 ± 0.75	0.00	13.08 ± 0.58	12.94 ± 1.24	28.50 ± 0.32
	16.23 ± 2.45	21.73 ± 0.81	17.04 ± 1.01	0.00	13.55 ± 0.94	12.87 ± 0.78	25.32 ± 0.67
	72.74 ± 3.67	11.21 ± 0.96	37.31 ± 2.56	0.00	62.70 ± 1.78	26.43 ± 1.78	30.94 ± 1.09
DX (μg·g^−1^)	56.35 ± 3.44	13.03 ± 0.74	35.06 ± 1.98	0.00	45.05 ± 1.56	18.74 ± 2.10	20.26 ± 0.72
	793.52 ± 12.55	72.09 ± 2.44	222.19 ± 2.78	1.60 ± 0.10	16.00 ± 0.89	35.70 ± 1.90	18.38 ± 0.30
	210.56 ± 7.23	30.81 ± 1.10	14.28 ± 0.51	0.75 ± 0.08	17.60 ± 1.04	52.82 ± 4.43	20.66 ± 1.14
	452.48 ± 9.97	43.81 ± 2.35	64.33 ± 1.77	0.88 ± 0.05	43.96 ± 1.13	86.96 ± 4.57	25.44 ± 0.28
DM (μg·g^−1^)	155.71 ± 9.73	33.09 ± 1.47	21.46 ± 0.88	0.42 ± 0.02	46.12 ± 0.67	158.82 ± 4.39	30.90 ± 0.40
	108.47 ± 6.98	24.90 ± 1.03	19.84 ± 0.45	1.03 ± 0.09	24.87 ± 0.77	97.37 ± 3.51	22.00 ± 1.01
	227.73 ± 10.34	15.04 ± 0.65	16.05 ± 1.30	0.62 ± 0.01	32.26 ± 1.46	38.62 ± 2.01	20.51 ± 0.61
	453.95 ± 11.11	44.92 ± 1.33	16.84 ± 1.31	0.59 ± 0.01	23.62 ± 1.11	24.35 ± 1.16	23.94 ± 0.63

Values in the table are means ± S.D.

Table 2 shows that the highest content of Cu, Zn, Pb, and Cd were found at DX, where Asia’s largest copper mine is located. Here, a large volume of acidic wastewater is produced every year, and most of the acidic wastewater is discharged into the Dawu river untreated, causing Dawu River, Le’an River, and Poyang Lake water to be involved in varying degrees of contamination and ecological damage. The highest content of As, Ni, and Cr were found in the JDZ area, which owns multiple industrial parks and has long been famous for its ceramic manufacturing industry. The content of all 7 metals in the UP area are low and the element Cd was below the detection limit (Table 2). This is because the UP area is a tourist attraction with minimal industrial, agricultural, and domestic pollution. The DM area has a more complicated situation: As seen from Table 2, the concentrations of elements Cu, Zn, Pb, and Cd are lower than the DX area. This could be likely due to the enrichment of heavy metals along the Le’an River. However, the elements Cr, As, and Ni show an increasing trend. The possible reason could be the proximity of this area to the downstream of Leping City, which has dense population, heavy traffic, and several factories such as electrical industry, cement, beer, paper, chemical products, etc.

The background values^19^ of heavy metals in Poyang Lake is shown in Table 3. It can be seen that the concentrations of the majority of elements in the surface sediments are greater than their background values. This is particularly prevalent for Cu, which is 41.52 times higher than its background value due to the famous copper mine. The enrichment degrees of As and Pb are 5.87 and 3.17 times, respectively. The other elements (Zn, Cd, and Cr) remain basically unchanged. Compared to the corresponding background concentrations in sediments of the Raohe Basin, the enrichment degree of seven metals increased in the following order: Ni < Cd < Zn < Cr < Pb < As < Cu.

**Table 3:** Background values and Multiple values in sediments along study area (μg·g^−1^).

	Cu	Zn	Pb	Cd	Cr	As	Ni
Mean values	197.21	32.31	39.63	0.51	35.26	78.52	31.03
Background^*^	4.75	45.75	12.5	0.75	29.65	13.37	—
Multiple	41.52	0.71	3.17	0.68	1.19	5.87	—

Background^*^: the background values of heavy metal in sediments from the Poyang Lake^19^.

### Heavy metal pollution assessment

#### Assessment Based on the Geo-accumulation Index

The *I*_*geo*_ was used to assess the pollution degree of heavy metals, and seven pollution grades were categorized according to the values of *I*_*geo*_: class 0: *I*_*geo*_ ≤ 0, uncontaminated; class 1: *I*_*geo*_ ≤ 1, uncontaminated to moderately contaminated; class 2: *I*_*geo*_ ≤ 2, moderately contaminated; class 3: *I*_*geo*_ ≤ 3, moderately to strongly contaminated; class 4: *I*_*geo*_ ≤ 4, strongly contaminated; class 5: *I*_*geo*_ ≤ 5, strongly to extremely contaminated; class 6: *I*_*geo*_ > 5, extremely contaminated^11^.

Table 4 shows that the range of averaged *I*_*geo*_ values obtained at different areas varies significantly. The Raohe Basin was heavily contaminated with heavy metal Cu, with averaged value of 3.75, reaching class 4. According to this table, the *I*_*geo*_ values of Cu fell into three different classes (class 3, 5, and 6), indicating the varying quality of sediments and the differences of local contamination. Especially in DX and DM areas, Cu has fallen to class 6 and class 5 respectively, suggesting strong to extreme contamination. Pb and As with *I*_*geo*_ ≤ 1 (uncontaminated to moderately contaminated) make up 50% of the samples, the rest are moderately contaminated (*I*_*geo*_ ≤ 2). *I*_*geo*_ of Zn, Cd, and Cr showed that all sampling areas fell in the uncontaminated class (*I*_*geo*_ ≤ 0), which could be deemed uncontaminated with regard to the tested metals.

**Table 4:** Geo-accumulation index (*I*_*geo*_) of the monitored trace metals.

Mean values (class)	JDZ	UP	DX	DM	Average (class)
Cu	2.68 (3)	2.30 (3)	5.16 (6)	4.84 (5)	3.75 (4)
Zn	−0.81 (0)	−1.99 (0)	−1.03 (0)	−1.33 (0)	−1.29 (0)
Pb	0.23 (1)	0.38 (1)	1.46 (2)	−0.03 (0)	0.51 (1)
Cd	−1.20 (0)	—	−0.15 (0)	−0.84 (0)	−0.55 (0)
Cr	−0.05 (0)	−0.99 (0)	−0.70 (0)	−0.54 (0)	−0.57 (0)
As	2.94 (3)	−0.29 (0)	1.06 (2)	1.62 (2)	1.33 (2)
Ni	—	—	—	—	—

Table 4 also shows that the DX sampling area, where the mining activities produce a large amount of wastewater, had the highest values of *I*_*geo*_ for Pb and Cu, which fell into class 2 and 6, respectively. Between JDZ and UP sampling areas, there were not much difference in *I*_*geo*_ values for Cu, Zn, Pb, and Cr. However, the largest *I*_*geo*_ value of As was 2.94, found at the JDZ sampling area, which far exceeded that UP. Coal and ceramic industries are developed at JDZ, especially ceramics is an important part of the economy at JDZ, which will inevitably impact the environment as a result of the burning of coal for the production of ceramic. The smallest *I*_*geo*_ values for most metals were obtained at the UP sampling area, where industrial, agricultural, and domestic pollution sources were minimal. Here, the *I*_*geo*_ values for Zn, Pb, Cd, Cr, and As were all ≤1. Only Cu could be considered as showing moderate to strong contamination with a mean *I*_*geo*_ value of 2.30. According to the averaged *I*_*geo*_ values, the pollution degree of heavy metals in sediments followed the order of: Cu > As > Pb > Cd > Cr > Zn.

#### Comparison with sediment quality values

In the present research, the marine sediment values for SQUIRT were introduced. SQVs has been widely used in freshwater and marine ecosystems to assess the sediment quality and to detect contaminations of aquatic ecosystems. Comparing the means of total concentrations of heavy metals in sediments to PEL (Table 5) shows that all the sampled sediments lay below PEL except for Ni and As in the JDZ area, as well as Cu and As in both the DX and DM areas. Available from Table 2, the averaged concentrations of As and Ni at JDZ were 177.89 and 55.83 μg·g^−1^ respectively and the averaged Cu and As concentrations at DX and DM were 378.23, 48.56 μg·g^−1^ and 236.46, 79.79 μg·g^−1^, respectively. Most of them had exceeded ERM, especially As at JDZ and Cu at DX; their values were higher than ERM and exceeded 100 μg·g^−1^, which suggested that any organisms that lived in these sediments would likely be adversely affected. However, by comparing the maximum value of total heavy metal concentration to the SQUIRT guidelines (Table 5), sediments from the JDZ sampling area would lie above PEL for As and Ni, sediments from the DX sampling area would lie above PEL for Cu, Pb, and As, and the maximum values as well as the means of the concentrations of Cu and As in the DM sampling area would lie above PEL.

**Table 5:** Screening Quick Reference Table^21^ for trace metals in marine sediments (μg·g^−1^).

	TEL	ERL	PEL	ERM	AET
Cu	18.70	34.00	108.00	270.00	390.00 (MO)
Zn	124.00	150.00	271.00	410.00	410.00 (I)
Pb	30.24	46.70	112.00	218.00	400.00 (B)
Cd	0.68	1.20	4.21	9.60	3.00 (N)
Cr	52.30	81.00	160.00	370.00	62.00 (N)
As	7.24	8.20	41.60	70.00	35.00 (B)
Ni	15.90	20.90	42.80	51.60	110.00 (EL)

End points of bioassay: B: Bivalve, E: Echinoderm larvae, I: Infaunal community impacts, L: Larval bioassay, M: Microtos, N: Neanthes, O: Oyster larvae.

**Figure 2:**
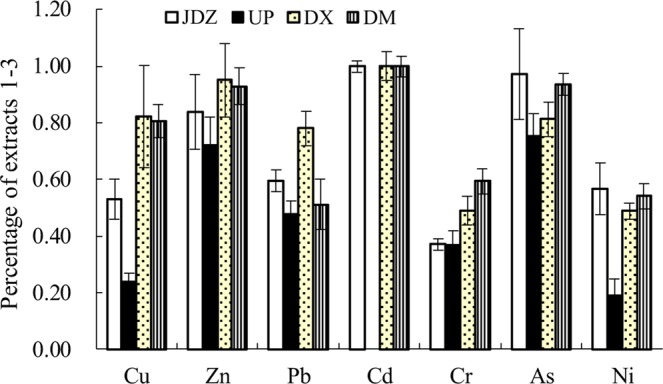
Metals extracted from fractions 1–3 as a percentage (±S.D.) of total metal concentration.

The averaged partitioning data for sediments of JDZ, UP, DX, and DM are shown in Table 6. In the present research, increased mobility was estimated via calculating the percentages of extracts 1–3 in the total concentrations of heavy metals (Fig. 2). This may also provide an indication of potential bioavailability. Even if this method cannot accurately predict bioavailability, bioavailability correlates to chemical fractions more than to the total metal concentrations^20^. Stable metal fractions that are most likely unavailable were excluded as residual fractions. Figure 2 indicates that the potential bioavailability of heavy metals in these areas is high. The UP area has a relatively low percentage (partitioned fractions 1–3) due to the less perturbed nature and clean environment. However, except this area (UP), extracts (1–3) of almost all the elements constituted more than 50% of the total content. The potential bioavailability exceeds 80% especially for Zn, Cd, and As. Unlike UP area, most heavy metal loads in other areas come from external sources. This strongly demonstrates that the impact of human activities is significant.

**Table 6:** Partitioning values of metals (Raohe Basin, μg·g^−1^).

	Ext. 1	Ext. 2	Ext. 3	Ext. 4
**JDZ**				
Cu	22.38 ± 2.76	7.68 ± 0.58	1.23 ± 0.41	27.59 ± 3.61
Zn	11.90 ± 1.48	8.34 ± 1.03	13.44 ± 1.77	6.48 ± 0.30
Pb	1.20 ± 0.09	11.90 ± 0.87	0.30 ± 0.01	9.09 ± 1.13
Cd	0.38 ± 0.05	0.04 ± 0.00	0.00	0.00
Cr	2.77 ± 0.43	11.56 ± 1.65	4.80 ± 0.07	32.50 ± 4.77
As	52.57 ± 2.12	52.96 ± 2.75	67.60 ± 3.53	4.76 ± 1.19
Ni	6.99 ± 1.33	6.01 ± 0.57	18.62 ± 0.88	24.21 ± 3.34
**UP**				
Cu	4.07 ± 0.21	1.84 ± 0.23	4.01 ± 1.05	31.90 ± 2.29
Zn	3.24 ± 0.46	5.60 ± 1.24	4.16 ± 0.97	5.04 ± 0.58
Pb	0.15 ± 0.02	10.59 ± 0.76	1.53 ± 0.08	13.51 ± 2.00
Cd	0.00	0.00	0.00	0.00
Cr	1.00 ± 0.32	8.01 ± 2.48	1.96 ± 0.28	18.81 ± 2.13
As	1.65 ± 0.11	4.40 ± 0.37	7.08 ± 1.34	4.28 ± 1.02
Ni	0.48 ± 0.08	0.66 ± 0.08	4.24 ± 0.69	22.88 ± 4.30
**DX**				
Cu	168.30 ± 4.59	73.79 ± 5.34	69.38 ± 4.27	66.75 ± 2.08
Zn	17.96 ± 1.44	12.69 ± 1.57	7.34 ± 1.22	1.94 ± 0.53
Pb	18.32 ± 1.26	42.17 ± 5.05	4.99 ± 1.35	18.48 ± 2.54
Cd	0.77 ± 0.11	0.00	0.04 ± 0.00	0.00
Cr	1.27 ± 0.21	10.29 ± 0.81	3.43 ± 0.35	15.66 ± 0.97
As	8.13 ± 1.27	8.46 ± 1.11	22.84 ± 1.73	9.12 ± 2.02
Ni	1.52 ± 0.33	2.24 ± 0.30	6.58 ± 1.07	10.85 ± 1.49
**DM**				
Cu	128.71 ± 5.79	24.09 ± 3.38	37.74 ± 2.19	45.93 ± 4.10
Zn	11.82 ± 0.77	5.48 ± 0.11	10.06 ± 1.47	2.12 ± 0.17
Pb	1.44 ± 0.44	7.80 ± 1.45	0.24 ± 0.06	9.07 ± 1.67
Cd	0.66 ± 0.14	0.00	0.00	0.00
Cr	2.72 ± 0.50	12.79 ± 2.01	3.31 ± 0.72	12.90 ± 1.58
As	28.67 ± 4.14	16.70 ± 2.53	29.30 ± 2.67	5.12 ± 0.63
Ni	3.21 ± 0.80	2.15 ± 0.13	7.80 ± 1.88	11.18 ± 2.09

Values in the table are means ± S.D.

Comparing the concentration of metals in the bioavailable fractions with the standards listed in SQUIRT showed that the concentration of As at all sampling areas was higher than ERL. The concentrations of the bioavailable fractions of Zn and Cr at all sampling areas were lower than the TEL, which might be considered as uncontaminated as for these two metals. The concentration of bioavailable fractions of As at JDZ even exceeded ERM, while Zn, Pb, Cd, and Cr was below the TEL. Apart from As, the concentrations of metals (extracts 1–3) at UP were lower than the TEL, indicating that no adverse biological effects were likely to occur. The concentrations (fractions 1–3) of Zn, Pb, Cd, Cr, and Ni at the DM sampling area were lower than the TEL, but the concentrations of Cu was above the PEL, and As was above the ERM. At the DX sampling area, the concentrations (fractions 1–3) of Pb and As exceeded ERL. It has been published that the incidence of effects increased about 10% for most trace metals while their concentrations were between ERL and ERM^15^. Moreover, the strongest risk to biota was found at the DX sampling area, where the concentrations (extracts 1–3) of Cu far exceeded ERM; here, adverse effects can always be expected. These findings indicated that a full environmental risk assessment could be completed.

## Conclusions

The present study set out to explore the heavy metal pollution in Jingdezhen Industrialized Area (JDZ), Upstream Area (UP), Dexing Mining Area (DX), and Downstream Area (DM) of Raohe Basin, Poyang Lake. A considerable spatial variation was found in the concentration of heavy metals. Most of the investigated areas (except UP) are heavily polluted due to anthropogenic activities essentially dominated by mining activities and industrial pollution. The mining activities have caused DX Area the highest concentrations of elements Cu, Zn, Pb, and Cd, while the industrial pollution at JDZ Area led to the highest concentrations of the rest elements (As, Cr, and Ni). From an overall perspective, Raohe Basin has been strongly contaminated (class 4) by element Cu, with an enrichment degree of more than 40 times. As and Pb are also the dominant elements in the heavy metal pollution. Besides, the potential bioavailability of these heavy metals is very high, reaching up to 80%. These factors result in the fact that any organisms living in the sediments would likely be adversely affected. This is especially prominent in the mining area (DX) that has been extremely contaminated (class 6) as a result of wastewater discharge. The migration of heavy metals would lead to the same situation at downstream areas. This research has presented a comprehensive analysis of the heavy metal pollution and potential risk within an important water source. The results reported in this paper provide data support for heavy metal morphology and risk research in Poyang Lake, which may have certain theoretical significance for heavy metal pollution control and contribute to future monitoring research.

## Data Availability

The datasets analysed during the current study are available from the corresponding author on reasonable request.
